# Medical Communication Perceived Self-Efficacy (ME-CO) Scale: Construction and Validation of a New Measuring Instrument from a Socio-Cognitive Perspective

**DOI:** 10.3390/ejihpe12070056

**Published:** 2022-07-11

**Authors:** Vincenza Capone

**Affiliations:** Department of Humanities, University of Naples Federico II, 83100 Naples, Italy; vincenza.capone@unina.it

**Keywords:** communication self-efficacy, social perceptions and cognition, scale validation, hospital doctors, doctor–patient communication, patient–doctor relationship

## Abstract

The study presents the validation of a scale measuring physicians’ efficacy beliefs about their ability to manage issues related to communication with patients. Specifically, the tool focused on three fundamental phases of the clinical interview: collecting information, returning information to patients, and creating and maintaining a relationship with them. The research included two studies. Study 1 generated an item pool based on the literature review and developed a self-report questionnaire administered to a pilot sample of 150 physicians (M_*Age*_ = 49.36; SD = 1.98). The responses were subjected to exploratory analysis. In total, 636 physicians (M_*Age*_ = 47.99; SD = 8.68) took part in Study 2. Exploratory and confirmatory analyses yielded a final version of the tool consisting of an eight-factor structure with 31 items. Findings provided evidence of the robust psychometric properties of the scale and its usefulness in assessing physicians’ self-efficacy and defining effective interventions aimed at strengthening the doctors’ communication skills. The scale detected different aspects of physicians’ communication self-efficacy (asking questions, active listening, giving information, communicating an inauspicious diagnosis, non-verbal communication, recognition of patient’s clues and suggestions, information checking, and empathy).

## 1. Introduction

An adequate diagnosis and targeted pharmacological treatment are crucial elements in the medical profession. However, physicians’ effectiveness is powerfully shaped by proper communication [1,2,3], to the point of affecting the patient’s satisfaction with care. Physicians’ ability to provide transparent information and explanations to patients, the vocabulary they adopt, their proxemics, their availability, and their relationship with users affect the patient’s understanding of the disease and their satisfaction. Many studies have shown that doctors’ communication skills are a major factor in patient outcomes and nurturing patient satisfaction [4]. Physicians talking to patients reduces patient anxiety and increases patient satisfaction [5]. In addition to the patient, good communication can also benefit the doctors; it makes them satisfied with their job and helps them reach a proper diagnosis sooner [2,6,7]. Recently, communication has been given significant attention in attaining high-quality healthcare services [8]. On the topic, a large amount of research identified the characteristics of a good communicator [9]. In particular, the value of the affective and moral dimension in medical practice has been highlighted, paying attention to compassion, empathy, reliability, respect, honesty, and integrity but also interviewing skills, communication of diagnosis, and active listening skill. Therefore, effective communication is an essential factor associated with increased performance in health professionals [10]. It is well acknowledged that clinicians can gain communication skills [11,12] if they consider communication as a critical factor in determining better performance [13]. Despite this, few studies have examined communication self-efficacy beliefs in the relationship between doctor and patient [6]. Such beliefs are one of the most robust antecedents of behavior [14]. When individuals do not believe they have a chance of succeeding in a specific area of their lives, they are unlikely to engage in behaviors linked to this area. Furthermore, they do not use abilities they do not know they have. In line with the above, Lent and colleagues [15] conceived self-efficacy as one of the main cognitive processes of self-regulation, which affects both initial choices about a specific activity and its outcomes.

Consequently, analyzing physicians’ perceived self-efficacy in communication is the first step in evaluating their relationships with patients. The socio-cognitive theory considers the mind an emergent system characterized by large degrees of initiative and autonomy [16]. Indeed, the mind does not just react to the external and inner world’s stimuli but transforms them through processes and structures able to integrate experience and direct conduct according to personal goals. As a result, the unity, coherence, and continuity of the individual’s personality are ensured [17]. Within this theoretical framework, self-efficacy is conceived as the individuals’ belief in their capacity to execute behaviors necessary to reach specific results [14]. Individuals with higher self-efficacy feel more capable of achieving mastery in particular activities, situations, or aspects of their psychological and social functioning [17], showing, at the same time, more significant commitment and perseverance in carrying out their actions. The conviction of being able to dominate such situations leads them to perceive difficulties as opportunities to test and improve themselves.

In healthcare, it has been demonstrated that self-efficacy beliefs strongly affect physicians’ behavioral practices [18,19]. Nevertheless, few studies have specifically examined communication self-efficacy between health professionals and patients [1]. For example, Hyman and colleagues [20] investigated the role played by self-efficacy in physicians’ ability to counsel patients on health-related factors—such as high cholesterol or drugs—and found that physicians with low levels of self-efficacy spent little time on such issues. Following the same reasoning, Gramling et al. [21] investigated the role of general practitioners’ self-efficacy in recommending preventive screening for patients with a family history of cancer. The authors documented that, although doctors believed that screening for hereditary cancer risk was necessary, they did not feel able to communicate this adequately to the patient, leading them not to recommend such protective behavior. So, the possession of knowledge and skills is not sufficient to achieve high performance if there is a lack of confidence about their ability to use them appropriately. Hence, training targeting knowledge is not enough to lead to significant changes. Effective interventions to improve health care need to clearly define roles and responsibilities and address clinicians’ skills in promoting patients’ behavioral change [22].

A study on the determinants of health care professionals’ communication behaviors with terminally ill cancer patients [23] highlighted that knowledge, skills levels, self-efficacy, outcome expectations, and psychological and social support were essential for effective communication. By contrast, low practical skills and self-efficacy, outcome expectancy beliefs, and lack of support may increase physicians’ anticipatory anxiety and adoption of self-protective coping styles, such as distancing and avoidance. Thus, health professionals with adequate skills, great confidence in their ability to succeed, and positive outcome expectations for patients and themselves are more facilitated in doctor–patient communication and more willing to enhance their skills.

Concerning the differences in communication self-efficacy among different health professionals, some studies [2,24] reported that physicians had a higher level of communicative self-efficacy than nurses. On the other hand, no significant differences were found according to age for both categories and professional seniority. In addition, higher levels of communication self-efficacy were associated with lower stress levels.

Although there are studies on communication self-efficacy, the literature on the measurement instruments is still scarce. In some studies [18], the authors did not present the scale. In Gramling and colleagues’ [21] work, the tool adopted to assess the efficacy beliefs of family physicians consisted of a few very specific items. The outdated study of Parle and colleagues [23] referred to the self-efficacy of oncologists to communicate with terminally ill patients, detected with an instrument developed ad hoc. Therefore, this tool is not very compatible with the purpose of research in other areas of healthcare. Recently, Feldman and colleagues [25] also developed a scale to measure several communication skills, including “disclosing difficult news in manageable chunks so that the patient is not overwhelmed” and “determining how to present information according to the patient’s emotional state”. However, similar to Parle and colleagues’ [23] scale, it was designed for the oncology context only.

## 2. The Current Study

### Aim and Hypotheses

The primary purpose of this work was to develop an original tool to evaluate physicians’ beliefs regarding their ability to cope with topical situations related to doctor–patient communication. Consequently, the specific objectives were to construct and validate the Perceived Self-efficacy in Medical Communication Scale (ME-CO) in the hospital context.

It must be underlined that the scale aims to measure the hospital doctors’ effectiveness in communicating with a patient they have already met at least once. This scale characteristic results from the substantial amount of literature on the first encounter between a physician and patient [26,27]. Such literature supports the uniqueness of this clinical interview from all others and highlights how various obstacles and patient profiles shape it. The level of generality of the scale can be considered, hence, as intermediate.

No assumptions were made regarding the scale factorial structure and internal consistency. However, the instrument validity analysis was guided by some general hypotheses. Specifically, since the scale was developed based on the literature in this field, good content validity was assumed. In addition, it was hypothesized that the implementation of exploratory (EFA) and confirmatory factor analyses (CFA) should have yielded the existence of a single latent psychological construct that could have explained the scale scores. Regarding concurrent validity, we expected a positive and significant correlation between the ME-CO Scale and the Perceived Social Self-efficacy Scale [28]. The latter scale was chosen based on a series of evaluations regarding the social nature of communication; being able to communicate effectively with a patient is not indifferent to the extent to which physicians feel able to establish collaborative relationships, take the initiative, represent their points of view, and carry out a functional relationship with another person. Given the characteristics of a communicative act and the variety of interpersonal relationships that one deals with daily, it is plausible to suppose a range of different abilities, which led us to hypothesize a correlation between the two scales.

In light of the above, two studies were carried out as follows:

Study 1—Phase I: Development of the ME-CO Scale through building an item pool based on literature exploration consistently with Bandura’s recommendations [29].

Study 1—Phase II: Analysis of the psychometric properties of the first version of the scale (pilot study) with a sample of physicians.

Study 2—Analysis of the psychometric properties of the final version of the scale by using a larger sample of physicians.

## 3. Materials and Methods

### 3.1. Development of the Preliminary Version of the Scale

In order to develop the ME-CO Scale, the literature on communication was explored, focusing on several essential aspects: (a) the definition of effective medical communication [30], (b) the skills and strategies proposed by the patient-centered approach [31], and (c) the literature on communication skills in the medical field [32,33,34,35,36]. However, these aspects were not sufficiently exhaustive, since the self-efficacy construct specificity did not allow the systematic application of the studies’ findings to the Italian context. A recognition of the challenges, obstacles, and critical situations specific to the hospital environment was needed. Consequently, a preliminary study was conducted through a qualitative methodology, involving hospital doctors whose point of view allowed us to identify the patients’ profiles with whom it is more challenging to establish effective communication and the contextual obstacles affecting it [37]. The areas considered for the scale construction referred to the three non-sequential phases of the doctor–patient communication: the information gathering, return of information to the patient, creation and maintenance of the relationship [38]. Moreover, the essential skills for effective communication implementation in each of the mentioned phases were taken into account. In the information gathering phase, various skills are needed: *questioning skills* [39]—doctor’s ability to ask questions to know the patient’s point of view; *prompts and cues skills* [33], which consist of the ability to recognize and use the clues and the patient’s suggestions with the aim to encourage the expression of the underlying information; and *active listening skills* [34], which refer to the use of silence, encourage the patient to keep talking, the time dedicated to him/her, and the respect of the speech turn.

In the restitution of the information phase, we identified the role of *checking skills* [40], which consists of the checking of the information given and received by the patient; the *communication skills* in sharing the diagnosis [41], which represents the ability to clearly explain to the patient which pathology affects him/her; and the *talking skills* [32] or the ability to speak to the patient properly, using an understandable language in providing medical information, without technical terms and following a logical sequence in the exposition of contents.

Finally, the relationship maintenance phase requires *reflection skills*, which consist of an empathic way of returning to the patient information that is linked to him/her agenda, in particular: his/her feelings; the ability to be empathic by eliciting the patient’s feelings, using paraphrases in repeating what the patient says; the use of silence and the interest in listening to what the patient struggles to share; the ability to manage the “non-verbal” language [42,43], a skill that consists of knowing how to manage and control their gestures adequately and especially in “holding” the patient’s gaze [31].

The building of ME-CO Scale items resulted from the integration of elements present in the literature with those that emerged from the qualitative study [37]. For instance, regarding checking skills, the literature [31] suggested that such competencies may translate into the use of paraphrases and short summaries during doctor–patient communication. Hence, in the item formulation, we considered these activities, and consequently, a specific obstacle to communicative effectiveness was proposed to the doctors in line with what emerged from the interviews. Specifically, physicians entrusted information checking to their intuition rather than accurately verifying it. The relative item, therefore, was formulated as follows: “*How much do you feel able to check through direct questions whether the patient has understood the diagnosis, even if it seems from the patient’s statements that he/she has understood it?*”. For each of the skills mentioned above, specific tasks and difficulties were identified, resulting in the formulation of the items (Table 1).

### 3.2. Participants and Procedures

In this first study, the scale was administered to a reduced sample of physicians, following the suggestions of Osterlind [44]. In implementing a pre-test study, the sample should include at least 50–100 participants to identify the most problematic and difficult-to-understand items, assess the instructions’ effectiveness, and verify any errors in the presentation of the instrument.

The pilot study involved 150 physicians (74.7% men) aged 30–63 years (M = 49.36; SD = 1.98) who answered a self-report questionnaire. They worked in various hospitals in Campania, a region in Italy. Most of them carried out top-level roles in the hospitals: 72.7% were first-level managers, 16.0% were second-level medical managers, 8.7% were service managers, and 2.7% were researchers. Approval for the current study was obtained from each head of the hospital department. Participation was voluntary and anonymous, and all participants provided informed consent. The questionnaire was completed in an average of 40 min.

In the second study, a larger sample of Italian hospital doctors working in public and private sectors in Campania and with different medical specializations were invited to participate. A total of 1800 physician received a personalized letter by regular postal service. Each envelope contained a consent form, a self-report questionnaire, and a self-addressed return envelope to be sent back to the research team. Questionnaires were anonymous and confidential and kept in a locked filing cabinet. The sample consisted of 636 physicians (response rate = 35.3%), mostly men (67.4%) aged 29 to 67 years (M = 47.99; SD = 8.68). A total of 60.4% of the sample were first-level managers, 11.9% were second-level medical managers, 12.3% were in charge of the service, and 16.4% carried other job positions (i.e., researcher, contract worker, doctoral student). Their professional seniority—the years spent in the medical profession—was, on average, 18.08 years (SD = 9.76). In addition, the average seniority in the same hospital was 13 years (SD = 9.39), and the average ward-related seniority was 10.19 years (SD = 8.49). The weekly working hours they spent in their wards were, on average, 36.80 (SD = 5.64). Participants’ medical specializations were as follows: general surgery (17.2%), cardiology (14%), gynecology and obstetrics (14.7%), anesthesia and resuscitation (5.1%), internal medicine (8.4%), neurology (7.5%), orthopedics (5.7%), radiology (3.3%), ophthalmology (2.9%), hematology (2.4%), diabetology (2.2%), dermatology (2.2%), and otolaryngology (2.2%). Specialists in psychiatry were not included in the sample because they use communication as a privileged tool for diagnosis and therapy. Likewise, pediatricians were excluded because the relationship with the patient is usually mediated by an adult, while the scale is designed for a direct relationship with the patient. Specialists in oncology and emergency medicine were also excluded due to their peculiarity specializations [45]. The questionnaire consisted of 80 items and was introduced by a general delivery in which, in addition to giving brief indications on the reasons for our work and guaranteeing the anonymity of the information, instructions were given on how to answer. The self-report instrument was administered to the doctors by a researcher specialized in health psychology and trainees in psychology who had received specific training on the research project and how to present it. The general and health directors of the hospital companies were contacted who authorized the administration of the questionnaire within the hospitals. Similar to Study 1, participation was voluntary, and the anonymity of participants was guaranteed. The average time for completion was 30 min.

### 3.3. Measures

In addition to demographic data and other data relating to the performance of the medical profession (years of service, years of work in the same hospital), the questionnaire included the following scales:

*The initial version of the ME-CO Scale.* It consisted of 64 items: 19 aimed at detecting self-efficacy beliefs during the information gathering phase, 23 concerning the phase of returning information, and 22 linked to the phase of building and maintaining the relationship. The items were assessed on a 5-point Likert scale from 1 (“*not at all able”*) to 5 (“*completely able*”). Participants were provided with the following instructions: “The statements in the questionnaire refer to some aspects of the doctor–patient interview that are sometimes difficult. Read the questions carefully and indicate the extent to which you feel able to deal with each situation described below by marking the number corresponding to your level of ability. There are no right or wrong answers. Imagine yourself in each of the situations described and answer truthfully”. The delivery, instead, was as follows: “During a hospital visit to one of your patients whom you have already met at least once, how much do you feel able to...”. Cronbach’s alpha was 0.98.

*Measure for concurrent validity: The Perceived Social Self-efficacy scale* [28]. This is a 15-item scale that detects the individuals’ beliefs relating to their ability to fit easily, feel at ease, and play a proactive role in social situations. It consists of 15 items. Participants indicated their level of agreement on a 5-point Likert scale (1 = *“definitely disagree”* to 5 = *“definitely agree”*). A sample item was “How capable are you of…”: “expressing your own opinion when, in the company of your friends, you are discussing something”, “working in a team”. For the present study, α was 0.96.

In Study 2, the questionnaire included, in addition to items for collecting demographic data, the 55-item ME-CO version and the Perceived Social Self-efficacy Scale [28] already used in the first study (α = 0.95).

### 3.4. Statistical Analyses (Study 1 and Study 2)

The ME-CO items were evaluated with regard to variance and frequency distribution as a means of selecting the appropriate ones to be used in factor analysis (study 1). The Kaiser–Meyer–Olkin (KMO) measure of sampling adequacy and Bartlett’s test of sphericity were used to test whether the dataset was appropriate for factor analysis.

The dimensionality of the scale was investigated using exploratory factor analysis (study 1 and study 2). In order to facilitate the interpretation of the factor analysis, we followed the recommendations of Fabrigar and colleagues [46] and performed a principal-axis factor analysis with Promax rotation, hypothesizing the high relation between factors [47]. In order to calculate reliability, we used the analysis of internal consistency through covariance between items using Cronbach’s alpha, an index recommended by Bandura [13]. In order to analyze internal consistency, we calculated the corrected correlation between the score of the item and the total scale [47,48,49]. Confirmatory factor analysis (CFA) was conducted, using the maximum likelihood estimation method, to evaluate the underlying structure of items (study 2). The full sample of participants in study 2 was randomly split into two halves to increase the stability of our results. The second sub-sample was used to verify the former solution, implementing a CFA model. We used the Mplus 8.0 computer program to perform the analyses. Each first-order factor was measured by items identified in the EFA, and no cross-loadings were allowed. In order to evaluate the solution, we took into account different goodness of fit indices: X2, root mean square error of approximation (RMSEA), root mean square residual (RMSR), comparative fit index (CFI), and the Tucker–Lewis index (TLI). CFI and TLI values above 0.90 [50,51,52], RMSEA values below or equal to 0.06, and SRMR values equal to or below 0.09 [52] were considered adequate.

## 4. Results

### 4.1. Results of Study 1

The mean scores of the responses to the 64-item ME-CO version ranged between 4.30 and 2.91, whereases the standard deviations were between 1.15 and 0.75. The analyses of skewness and kurtosis did not reveal values that would have led us to eliminate any item. Bartlett’s sphericity test was equal to *X*^2^ (*df* =2016, *N* = 150) = 7665.35, *p* < 0.000, and the Kaiser–Meyer–Olkin index (KMO) was 0.91, indicating an adequate correlation matrix for factor analysis.

Scale dimensionality was investigated through the exploratory factor. The analysis revealed 11 factors with an eigenvalue greater than 1, which explained 61.67% of the total variance. Nine items were eliminated based on such analysis, since they were characterized by a saturation lower than 0.30 or with double saturation [48]. As a result, the items were reduced to 55. The items eliminated were the following: 17, 31, 55, 10, 48, 43, 39, 52, 38. Cronbach’s alpha was computed to verify the scale reliability [29]. The reliability coefficient for the entire 64-item scale was very high (α = 0.98). A 55-item solution was tested, reporting a high Cronbach’s alpha equal to 0.95. Finally, we recalculated Bartlett’s sphericity test, which was equal to *X*^2^ (*df* = 1540, *N* = 150) = 7665.35, *p* < 0.000. Moreover, the KMO was 0.88. The 11-factor structure explained 66.99% of the total variance. The findings of the bivariate correlation analysis showed that the 55-item ME-CO version had a high correlation with the Perceived Social Self-efficacy (*r* = 0.65; *p* < 0.001), reporting a good concurrent validity.

### 4.2. Results of Study 2

#### 4.2.1. Descriptive Analyses

Hospital doctors reported high average levels of perceived self-efficacy in medical communication (3.60) as well as in all sub-dimensions (Table 2). Moreover, the respondents showed relatively high value of perceived social self-efficacy (3.28); thus, they considered themselves capable of easily fitting into a group and playing a proactive role in social situations.

Respondents were grouped according to their professional seniority defining the following classification:

(1) Young physicians: 1 to 9 years in the medical profession (N = 190).
(2) Career professionals: 10 to 20 years in the medical profession (N = 218).
(3) Veterans: 21 to 44 years in the medical profession (N = 222).

The analysis of univariate variance (Table 3) and Tukey’s post hoc test showed that veterans had higher levels of self-efficacy in communication with patients than young physicians and career professionals (veterans = 3.92; young physicians = 3.62; career professionals = 3.61). Specifically, it was found that veterans had higher levels of self-efficacy in providing clear information to patients than young physicians (veterans = 4.14; young physicians = 3.88; professionals = 4.02). The same differences between groups emerged in the “Asking questions” factor (veterans = 3.83; young physicians = 3.60; career professionals = 3.78). Regarding the “Information checking” factor, the veterans and the professionals felt more effective than the young physicians (veterans = 3.53; professionals = 3.48; young physicians = 3.28). No differences were found between the groups regarding perceived social self-efficacy.

#### 4.2.2. Analysis of Psychometric Properties of the 55-Item ME-CO Version

The items of the ME-CO (55 items) showed a good approximation to the normal distribution (Table 4). The mean of the items ranged between 2.78 (item 64) and 4.10 (item 25). The standard deviation ranged from 0.80 (item 58) to 1.08 (item 28).

The correlation between the items’ scores and those of the total scale was calculated. The coefficients ranged between 0.42 (item 28) and 0.67 (item 53) and were considered adequate, since they were greater than 0.30 [47]. The KMO index was 0.95, and Bartlett’s sphericity test was *X*^2^ = 18705.02, *p* < 0.0000 (*df* = 1485, *N* = 636).

The exploratory factor analysis was performed. Considering eigenvalues greater than 1, the appropriate solution was found for the eight-factor one. A total of 24 items with double saturation or lower than 0.30 were eliminated: 12, 18, 30, 26, 29, 32, 61, 33, 34, 28, 40, 41, 47, 49, 50, 51, 60, 59, 58, 57, 42, 56, 54, 53. The analyses were repeated with the remaining 31 items. The KMO index was 0.94, and Bartlett’s sphericity test was *X*^2^ = 17,705.02, *p* < 0.0000 (*df* = 1455, *N* = 636). The final version supported the eight-factor structure. This solution seemed adequate, since, in the reproduced correlation matrix, only a few residuals (1%) were greater than 0.05 [49]. The eight factors explained 69.98% of the total variance. No item had saturations higher than 0.30 on more than one factor. Moreover, the scores of the item-total correlation and those of Cronbach’s Alpha if the item was eliminated suggested not to eliminate additional items (Table 5). The eight factors that emerged were defined as follows: self-efficacy in providing clear information (λ= 10.62), active listening (λ = 2.48), communicating a diagnosis (λ = 2.04), recognition of patient’s clues and suggestions (λ = 1.66), non-verbal communication (λ = 1.46), asking questions (λ = 1.27), being empathetic (λ = 1.12), and information checking (λ = 1.09).

Cronbach’s alpha of the 31-item ME-CO version was 0.93. Reliability was also verified for each of the eight dimensions that emerged from the factor analysis; reliability coefficients ranged from 0.77 to 0.90 (Table 3).

In order to evaluate the validity of the 31-item ME-CO version, exploratory and confirmatory factor analyses were carried out. The entire sample of participants was randomized and divided in half to increase the stability of the results.

#### 4.2.3. Exploratory Factor Analysis of the 31-Item ME-CO Final Version

Exploratory factor analysis was implemented on the first sample (N = 314) to test the appropriateness of the eight-factor structure. The eight-factor solution explained 69.98% of the total variance. The eigenvalues and factor loading supported the same factors (Table 6) that resulted from the previous analyses: self-efficacy in providing clear information (λ = 10.62), self-efficacy in active listening (λ = 2.48), communicating a diagnosis (λ = 2.04), recognition of patient’s clues and suggestions (λ = 1.66), non-verbal communication (λ = 1.46), asking questions (λ = 1.30), being empathetic (λ = 1.11), information checking (λ = 1.10).

#### 4.2.4. Confirmatory Factor Analysis of the 31-Item Final Version of the ME-CO Scale

**Figure 1:**
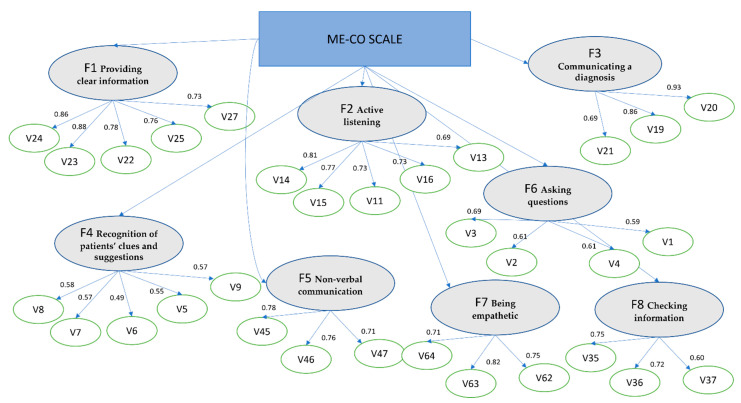
Confirmatory factor analysis—Standardized coefficients of 31-item ME-CO final version. Note: ME-CO by F1 (0.69); F2 (0.80); F3 (0.57); F4 (0.85); F5 (0.76); F6 (0.83); F7 (0.54); F8 (0.69).

The second sub-sample (N = 322) was used to verify the solution that previously emerged through the exploratory factor analysis. Thus, a confirmatory analysis model with eight first-order factors and one second-order factor—called ME-CO—was tested. In the model considered (Figure 1), the Chi-Square was 833.145 (*df* = 454 and *p* < 0.000); the CFI was 0.93, and the TLI was 0.92, the RMSEA = 0.05 (0.049 0.060; probability RMSEA ≤ 0.05 = 0.77) and the SRMR = 0.048.

#### 4.2.5. External Validity of the ME-CO Scale

A correlation analysis was carried out between the ME-CO Scale and its eight dimensions and the Perceived Social Self-Efficacy Scale to evaluate its external validity. The results of the correlation analysis show a good correlation between the ME-CO Scale and its subdimensions with the Perceived Social Self-Efficacy Scale, supporting the concurrent validity of the tools (Table 7).

## 5. Discussion

Self-efficacy is well recognized as a robust antecedent of behaviors [14], including those related to the communication between physicians and patients [2,21,23]. Consequently, the present study focused on the communication between doctors and patients by developing an assessment tool for physicians’ self-efficacy in building proper communication with patients: the ME-CO Scale.

In order to achieve the goals mentioned above, an extensive theoretical review was carried out. The latter helped us identify the following key points: the definition of effective communication, skills in medical practice, and self-efficacy, as well as the measures of perceived self-efficacy in healthcare and the obstacles to proper communication between doctor and patient.

Consistently with the recommendations of several authors who developed measures of perceived effectiveness from a socio-cognitive perspective [14,16,29,53], a set of 64 items was designed to assess hospital doctors’ self-efficacy beliefs about the communication with patients. The 64-item ME-CO version was tested through a pilot study. The findings of the first study suggested the elimination of nine items. Hence, the 55-item version of the tools was administered, along with other measures, to a sample of 636 hospital doctors. Analyses of the structure and reliability of the scale led to developing the final version of the ME-CO, consisting of 31 items with an eight-factor solution. Such structure showed good internal consistency, supported by a confirmatory factor analysis too. The results of the analyses substantially proved the robust psychometric properties of the ME-CO. The amount of the explained variance, the factor loadings of the items, and Cronbach’s alphas values were very satisfactory. Likewise, the content, construct, and criterion validities were adequate. Specifically, the significant correlations between the ME-CO and the Perceived Social Self-efficacy attested to the accuracy of the problematic areas considered in the scale development. Regarding participants’ levels of self-efficacy in communicating with the patient, they reported, on average, high levels in line with those of social self-efficacy. The physicians involved, therefore, perceived themselves as effective communicators in their profession. Additionally, the findings grasped differences between the subgroups of physicians regarding self-efficacy in communication, suggesting that this belief was linked to participants’ professional seniority. In particular, physicians with seniority greater than 20 years expressed greater confidence in their communicative effectiveness than the other groups, especially about providing clear information to the patient, asking questions, and checking the information given and received by patients. This result aligns with the socio-cognitive theoretical framework that identifies the comparison process with the task derived from the experience as one of the primary sources of self-efficacy [14].

### Limitation of the Study

We acknowledge that the findings of this study should be considered in the context of some limitations, which can be addressed in future research. In particular, this study did not maintain an equal proportion of physicians among different specializations and genders. Diverse and equally proportioned data need to be collected in future research by translating and validating the scale in further languages, so that it may be adopted to assess physicians’ perceived self-efficacy in other contexts. Furthermore, caution should be exercised in evaluating the criterion validity of the ME-CO Scale, since it was assessed through correlation analysis between self-report measures [53]. Hence, it would need to determine the associations of the scale with non-self-report assessments and include behavioral measures, such as the characteristics of the interviews (e.g., setting, duration, number of patients per day). Moreover, it cannot be excluded that common method variance and social desirability biases, typical of self-report tools [54,55], could have played a role in the study results. Although anonymity may have partially reduced the role of this social desirability in data collection and a number of different techniques (e.g., not explicitly referring to the psychological nature of this study), future studies should take a longitudinal approach to test the reliability over time. Finally, since self-efficacy is “domain specific” [14], this instrument cannot be applied in domains other than hospital healthcare without proper adaptation. In any case, for non-medical health professions, some scales for communication self-efficacy in nursing are available [56,57].

## 6. Conclusions and Implications for Research

Notwithstanding the limitations mentioned above, the study provides a tool with strong psychometric properties also able to catch differences in physicians’ communication self-efficacy according to their professional seniority. Furthermore, the tool responds to the need to pay attention to the assessment in the medical field. As stated by Borgogni [58], assessing the extent to which physicians perceive to be effective in their medical practice is an essential step in the diagnostic process of designing interventions. Consistently with this line of reasoning, the ME-CO Scale, due to its agile use at the operational level and its psychometric characteristics, is a tool that can provide useful information on both the research side and in intervention designing by an accurate evaluation of structured physicians’ and specializing doctors’ effectiveness in establishing proper communication with patients. Specifically, such interventions may improve physicians’ effectiveness in the relationship with users, resulting in higher satisfaction of health professionals with their job and patients with health care.

In addition to replicating this study in other care contexts, we suggest several areas that critically explore alternative explanations to our results and suggest research worthy of pursuit. First of all, there is a need for research to take multilevel factors more fully into consideration. For example, future studies should examine the impact of collective efficacy (team level) on self-efficacy [59]. It would also be interesting to use the instrument to investigate efficacy beliefs related to performance and organizational culture. For example, a hospital culture focused on patient engagement [60] is expected to reinforce perceptions of self- efficacy.

It would also be interesting to study the doctor–patient dyad, analyzing how the relationship between doctor and patient is influenced by the doctor’s communicative effectiveness (in terms of, for example, adherence to treatment and satisfaction). Communication is often used as a container concept. In many studies, communication skills are assessed without further differentiation between the communication elements; therefore, the possibility of applying the entire scale or only subsets of items tapping specific factors separately can be of great interest in some studies [61].

Finally, the ME-CO scale can be applied optimally to assess potential communication

Problems prior to conducting major interventions, target interventions designed to enhance the perception of efficacy beliefs, and incorporate evaluation of communication efficacy as part of regular employee assessments [62].

## Figures and Tables

**Table 1:** The ME-CO Scale—Examples of items concerning specific skills in each area.

Skill	Definition	Example of Item
**Area 1: Information gathering phase**		
*Questioning skills*	Encouraging the expression of the patient’s point of view, identifying symptoms and information necessary to frame his/her condition from a clinical and psycho-social perspective, and using *trigger* questions appropriately	How much do you feel able to encourage a low-talking patient to express his/her expectations about the outcome of your encounter?
*Prompts and cues skills*	Recognizing and using the clues and the patient’s suggestions, promoting the expression of the underlying information	How much do you feel able to understand from the few hints given by the patient that he/she has difficulties in accepting the therapy?
*Active listening skills*	Listening carefully to the patient, managing non-verbal communication, respecting the turn to speak	How much do you feel able to listen carefully to the patient, even in the presence of colleagues who see this practice as a waste of time?
**Area 2: Feedback phase**		
*Talking skills*	Talking to the patient using a non-technical language	How much do you feel able to explain a clinical report, for example, a CT scan, in simple language?
*Communication of the diagnosis*	Communicating an inauspicious diagnosis	How much do you feel able to communicate an inauspicious diagnosis to a very sensitive patient?
*Checking skills*	Ensuring that the patient has understood the doctor’s words, testing whether he or she has understood him/her. Checking whether what the doctor understood matches the information provided by the patient	How much do you feel able to check through direct questions whether the patient has understood the diagnosis, even if it seems from the patient’s statements that he/she has understood it? How much do you feel able to repeat the information expressed by the patient synthetically at different times during the visit, even if the interview has already lasted a long time?
**Area 3: Relationship building and maintenance phase**		
*Non-verbal use*	Using facial expressions, gaze, and proxemic to put the patient at ease	How much do you feel able to look into the patient’s eyes while you are proposing an instrumental investigation that the patient does not agree with?
*Empathy*	Respecting and caring for the patient, empathically returning information taking into account the patient’s agenda, particularly the dimension of their feelings	How do you feel able to show “warmth” to a patient who has had a negative experience with one of your ward colleagues?

**Table 2:** Mean and standard deviations of the ME-CO scale dimensions.

ME-CO Factors	M	SD
ME-CO—Providing clear information	4.02	0.71
ME-CO—Active listening	3.62	0.77
ME-CO—Communicating a diagnosis	3.04	0.86
ME-CO—Recognition of patient’s clues and suggestions	3.61	0.64
ME-CO—Non-verbal communication	3.74	0.79
ME-CO—Asking questions	3.71	0.69
ME-CO—Being empathetic	3.16	0.78
ME-CO—Information checking	3.45	0.78

**Table 3:** Analysis of variance: significant differences by years in the medical profession (means).

	Young N = 190	Professionals N = 218	Veterans N = 221	F (df)	Sig.
ME-CO	3.62 a	3.61 a	3.92 b	3.84_(2, 630)_	0.02
ME-CO—Providing clear information	3.88 a	4.02 a b	4.14 b	5.04_(2, 630)_	0.00
ME-CO—Asking questions	3.60 a	3.78 a b	3.83 b	4.04_(2, 630)_	0.01
ME-CO—Information checking	3.28 a	3.48 b	3.53 b	4.52_(2, 630)_	0.01

Note: a and b identify Tukey’s test result graphically.

**Table 4:** Descriptive statistics of items of 55-item ME-CO version.

ITEM	M	SD	Ske	Kur	ITEM	M	SD	Ske	Kur
**v25**	4.10	0.84	−0.56	−0.31	**v15**	3.64	0.90	0.00	−0.65
**v24**	4.09	0.87	−0.60	−0.23	**v2**	3.62	0.84	0.05	−0.51
**v23**	3.98	0.89	−0.41	−0.78	**v34**	3.59	0.82	−0.04	−0.19
**v27**	3.94	0.86	−0.38	−0.40	**v29**	3.58	0.85	−0.01	−0.24
**v61**	3.88	0.93	−0.44	−0.41	**v7**	3.58	0.84	−0.06	−0.19
**v51**	3.87	0.88	−0.26	−0.72	**v4**	3.57	0.88	−0.05	−0.50
**v1**	3.84	0.92	−0.22	−0.72	**v18**	3.57	0.95	−0.07	−0.71
**v47**	3.84	0.89	−0.33	−0.46	**v14**	3.52	0.98	−0.11	−0.57
**v40**	3.84	0.88	−0.32	−0.58	**v57**	3.52	0.92	−0.11	−0.27
**v56**	3.83	0.96	−0.51	−0.21	**v16**	3.51	0.96	−0.13	−0.59
**v22**	3.82	0.94	−0.35	−0.54	**v36**	3.51	0.93	−0.24	−0.28
**v45**	3.76	0.89	−0.12	−0.65	**v53**	3.48	0.95	−0.03	−0.40
**v6**	3.74	0.80	0.10	−0.61	**v59**	3.48	0.83	0.05	−0.47
**v54**	3.74	0.85	−0.26	−0.26	**v58**	3.47	0.80	0.34	−0.30
**v49**	3.74	0.88	−0.18	−0.53	**v30**	3.44	0.96	−0.21	−0.45
**v26**	3.73	0.88	−0.19	−0.43	**v63**	3.44	0.88	0.13	−0.49
**v50**	3.73	0.86	−0.15	−0.55	**v9**	3.44	0.92	−0.05	−0.46
**v12**	3.73	0.90	−0.17	−0.63	**v5**	3.43	0.93	−0.02	−0.60
**v13**	3.73	0.89	−0.13	−0.79	**v32**	3.40	0.95	−0.14	−0.30
**v44**	3.73	0.90	−0.13	−0.58	**v37**	3.38	0.93	−0.09	−0.26
**v28**	3.72	1.08	−0.33	−0.88	**v33**	3.38	0.91	0.02	−0.22
**v60**	3.71	0.80	0.09	−0.71	**v35**	3.31	0.95	−0.06	−0.43
**v41**	3.70	0.93	−0.38	−0.24	**v62**	3.21	0.98	−0.04	−0.32
**v3**	3.70	0.86	−0.13	−0.39	**v19**	3.07	0.93	0.18	−0.28
**v8**	3.67	0.86	−0.13	−0.36	**v21**	2.88	0.94	0.14	−0.19
**v46**	3.67	0.91	−0.16	−0.52	**v20**	2.83	1.04	0.14	−0.40
**v42**	3.65	0.90	−0.18	−0.25	**v64**	2.78	1.08	0.20	−0.43
**v11**	3.65	0.97	−0.25	−0.48					

Note: Item numbers refer to the initial 64-item ME-CO version; Ske = skewness; Kur = kurtosis.

**Table 5:** Factor loadings of the 31-item ME-CO version, item-total correlations, and item reliability.

Item	Factor Loading	Item-Total Correlation	α if Deleted
**Factor 1. ME-CO—Providing clear information (α = 0.90)**			
v24	0.95	0.82	0.86
v23	0.89	0.81	0.86
v22	0.74	0.72	0.89
v25	0.73	0.72	0.88
v27	0.63	0.70	0.89
**Factor 2. ME-CO—Active listening (α = 0.86)**			
v14	0.97	0.76	0.81
v15	0.80	0.72	0.82
v11	0.67	0.64	0.84
v16	0.64	0.65	0.84
v13	0.58	0.64	0.84
**Factor 3. ME-CO—Communicating a diagnosis (α = 0.88)**			
v20	0.92	0.81	0.79
v19	0.87	0.80	0.80
v21	0.69	0.69	0.88
**Factor 4. ME-CO—Recognition of patients’ clues and suggestions (α = 0.77)**			
v8	0.89	0.61	0.71
v7	0.66	0.60	0.71
v6	0.50	0.52	0.74
v5	0.47	0.50	0.75
v9	0.42	0.61	0.71
**Factor 5. ME-CO—Non-verbal communication (α = 0.86)**			
v45	0.85	0.78	0.77
v46	0.82	0.73	0.81
v44	0.80	0.70	0.84
**Factor 6. ME-CO—Asking questions (α = 0.82)**			
v3	0.89	0.68	0.75
v2	0.67	0.68	0.75
v4	0.65	0.60	0.78
v1	0.54	0.58	0.79
**Factor 7. ME-CO—Being empathetic (α = 0.77)**			
v64	0.90	0.65	0.64
v62	0.69	0.60	0.70
v63	0.59	0.58	0.73
**Factor 8. ME-CO—Information checking (α = 0.78)**			
v36	0.87	0.67	0.64
v35	0.71	0.62	0.70
v37	0.55	0.56	0.76

**Table 6:** Means, standard deviations, skewness, kurtosis, item-total correlations, and item reliability of the 31-item ME-CO final version within the two sub-samples.

	Mean ^a^/^b^	Factor Loading ^a^/^b^	SD ^a^/^b^	Ske ^a^/^b^	Kur ^a^/^b^	Item-Total Corr. ^a^/^b^	α if Deleted ^a^/^b^
**1. ME-CO—Providing clear information (α ^a^ = 0. 90, α ^b^ = 0.90)**							
v24	4.102/4.081	0.93	0.85/0.89	−0.54/−0.65	−0.46/−0.05	0.81/0.83	0.86/0.86
v23	3.997/3.966	0.85	0.87/0.92	−0.38/−0.42	−0.77/−0.80	0.79/0.83	0.87/0.86
v25	4.124/4.078	0.77	0.81/0.86	−0.48/−0.62	−0.66/−0.06	0.72/0.72	0.88/0.89
v22	3.869/3.773	0.75	0.90/0.97	−0.29/−0.38	−0.70/−0.46	0.71/0.72	0.89/0.89
v27	3.914/3.972	0.67	0.85/0.86	−0.31/−0.44	−0.34/−0.42	0.72/0.68	0.88/0.89
**2. ME-CO—Active listening (α ^a^ = 0.86, α ^b^ = 0.86)**							
v11	3.706/3.586	0.88	1.00/0.95	−0.40/−0.10	−0.30/−0.61	0.63/0.65	0.85/0.84
v14	3.455/3.578	0.71	0.99/0.96	−0.01/−0.20	−0.73/−0.35	0.77/0.76	0.81/0.81
v13	3.729/3.726	0.70	0.87/0.91	−0.15/−0.11	−0.69/−0.86	0.66/0.62	0.84/0.85
v15	3.659/3.630	0.52	0.91/0.89	−0.03/0.02	−0.62/−0.67	0.73/0.71	0.83/0.82
v16	3.548/3.481	0.44	0.96/0.96	−0.17/−0.08	−0.45/−0.70	0.64/0.66	0.85/0.84
**3. ME-CO—Communicating a diagnosis (α ^a^ = 0.88, α ^b^ = 0.88)**							
v20	2.783/2.870	0.91	1.03/1.05	0.18/0.10	−0.35/−0.43	0.82/0.80	0.77/0.79
v19	3.003/3.134	0.84	0.90/0.96	0.26/0.09	−0.10/−0.40	0.79/0.80	0.80/0.79
v21	2.788/2.969	0.66	0.94/0.94	0.24/0.04	−0.05/−0.24	0.69/0.69	0.88/0.88
**4. ME-CO—Recognition of patients’ clues and suggestions (α ^a^ = 0.78, α ^b^ = 0.78)**							
v8	3.636/3.713	0.79	0.86/0.86	−0.15/−0.12	−0.30/−0.42	0.59/0.63	0.72/0.70
v7	3.552/3.603	0.75	0.83/0.85	−0.037/−0.09	−0.21/−0.16	0.63/0.57	0.71/0.72
v9	3.383/3.489	0.48	0.93/0.91	−0.09/−0.01	−0.29/−0.66	0.53/0.50	0.74/0.74
v6	3.748/3.742	0.47	0.77/0.83	0.30/−0.05	−0.97/−0.36	0.53/0.50	0.74/0.74
v5	3.390/3.469	0.41	0.93/0.92	0.04/−0.07	−0.67/−0.51	0.48/0.51	0.76/0.74
**5. ME-CO—Non-verbal communication (α ^a^ = 0.86, α ^b^ = 0.87)**							
v46	3.662/3.676	0.84	0.88/0.95	0.00/−0.28	−0.78/−0.34	0.75/0.72	0.80/0.82
v44	3.717/3.735	0.77	0.88/0.91	−0.03/−0.22	−0.72/−0.44	0.70/0.70	0.84/0.83
v45	3.707/3.820	0.77	0.90/0.87	−0.03/−0.20	−0.87/−0.38	0.77/0.78	0.78/0.76
**6. ME-CO—Asking questions (α ^a^ = 0.82, α ^b^ = 0.82)**							
v3	3.696/3.707	0.84	0.84/0.88	0.00/−0.25	−0.56/−0.25	0.66/0.70	0.77/0.73
v4	3.550/3.590	0.69	0.87/0.88	0.14/−0.22	−0.70/−0.27	0.63/0.58	0.78/0.78
v2	3.599/3.640	0.65	0.86/0.82	0.07/0.04	−0.57/−0.44	0.71/0.65	0.75/0.75
v1	3.825/3.851	0.61	0.96/0.88	−0.27/−0.15	−0.69/−0.81	0.58/0.58	0.81/0.78
**7. ME-CO—Being empathetic (α ^a^ = 0.80, α ^b^ = 0.81)**							
v64	2.764/2.786	0.83	1.10/1.06	0.22/0.18	−0.45/−0.41	0.50/0.66	0.59/0.61
v63	3.473/3.413	0.73	0.85/0.91	0.17/0.11	−0.42/−0.56	0.39/0.55	0.72/0.74
v62	3.235/3.189	0.70	1.00/0.97	−0.05/−0.03	−0.40/−0.22	0.63/0.59	0.43/0.70
**8. ME-CO—Checking information (α ^a^ = 0.80, α ^b^ = 0.81)**							
v37	3.408/3.358	0.74	0.98/0.89	−0.05/−0.15	−0.45/−0.05	0.56/0.56	0.74/0.78
v36	3.525/3.488	0.58	0.94/0.92	−0.20/−0.29	−0.46/−0.08	0.68/0.66	0.61/0.67
v35	3.332/3.289	0.35	0.95/0.95	−0.03/−0.10	−0.55/−0.31	0.58/0.66	0.72/0.68

Note: ^a^ = First sub-sample data (N = 314); ^b^ = second sub-sample data (N = 322); Ske = skewness; Kur = kurtosis.

**Table 7:** Correlations between ME-CO Scale and Perceived Social Self-Efficacy Scale.

	1	2	3	4	5	6	7	8	9
1. ME-CO	1								
2. Providing clear information	0.75 **	1							
3. Active listening	0.78 **	0.49 **	1						
4. Communicating a diagnosis	0.60 **	0.35 **	0.36 **	1					
5. Recognition of patients’ clues and suggestions.	0.80 **	0.56 **	0.57 **	0.42 **	1				
6. Non-verbal communication	0.70 **	0.55 **	0.47 **	0.34 **	0.49 **	1			
7. Asking questions	0.77 **	0.49 **	0.53 **	0.40 **	0.63 **	0.49 **	1		
8. Being empathetic	0.64 **	0.29 **	0.50 **	0.34 **	0.42 **	0.34 **	0.41 **	1	
9. Information checking	0.70 **	0.46 **	0.49 **	0.34 **	0.45 **	0.40 **	0.48 **	0.50 **	1
10. Social self-efficacy	0.53 **	0.31 **	0.39 **	0.38 **	0.43 **	0.33 **	0.39 **	0.47 **	0.40 **

Note: ** *p* < 0.001.

## Data Availability

The data presented in this study and the items of the scale are available on request from the corresponding author.
